# Clinical characteristics and somatic burden of patients with mucopolysaccharidosis II with or without neurological involvement: An analysis from the Hunter Outcome Survey

**DOI:** 10.1016/j.ymgmr.2023.101005

**Published:** 2023-09-08

**Authors:** Heather Lau, Paul Harmatz, Jaco Botha, Jennifer Audi, Bianca Link

**Affiliations:** a Yale School of Medicine, New Haven, CT, USA; b UCSF Benioff Children's Hospital Oakland, Oakland, CA, USA; c Takeda Pharmaceuticals International AG, Zurich, Switzerland; d Division of Metabolism, Connective Tissue Unit, University Children's Hospital Zurich, Zurich, Switzerland

**Keywords:** Mucopolysaccharidosis type II, Hunter Outcome Survey, Enzyme replacement therapy, Surgery, Neuronopathic phenotype, Non-neuronopathic phenotype

## Abstract

Approximately two-thirds of patients with mucopolysaccharidosis II (MPS II; Hunter syndrome) have neuronopathic disease, with central nervous system involvement; one-third have non-neuronopathic disease. This analysis of data from the Hunter Outcome Survey (HOS) compared the clinical manifestations and surgical and nonsurgical procedure history in patients with neuronopathic or non-neuronopathic MPS II. Prospective patients were identified in July 2018 in HOS for inclusion in this analysis as those with stable cognitive impairment status as assessed at 10 years of age and at a minimum of one follow-up visit at 11 to <20 years of age. Patients were stratified according to cognitive impairment status at 10 years into neuronopathic and non-neuronopathic groups, and clinical manifestations and surgical and nonsurgical procedure history were compared between the two groups. In total, 193 patients had cognitive impairment status assessments available (at 10 years and 11 to <20 years of age), 151 of whom had stable cognitive impairment status and were included; 100/151 (66.2%) were in the neuronopathic group and 51/151 (33.8%) in the non-neuronopathic group. The proportion of patients demonstrating manifestations by system organ class and the number of surgical and nonsurgical procedures per patient were broadly comparable in the neuronopathic and non-neuronopathic groups both before and after patients' 10th birthdays. The most common manifestations before patients' 10th birthdays, including facial features, joint stiffness and limited function, and hepatomegaly were reported in >80% of patients in both groups. For the neuronopathic and non-neuronopathic groups, the median [10th percentile, 90th percentile] number of different types of surgical and nonsurgical procedures per patient (3 [1, 6] and 3 [1, 7], respectively) and of all procedures per patient (4 [1, 10] and 5 [2, 11], respectively) before patients' 10th birthdays were similar, although the type of procedure may have differed. Thus, in the first two decades of life, patients with non-neuronopathic disease were found to have similar somatic manifestations to those of the neuronopathic group and undergo procedures for complications as often as those with neuronopathic disease.

## 1 Introduction

Mucopolysaccharidosis II (MPS II; Hunter syndrome; OMIM 309900) is a rare, X-linked life-limiting disease caused by deficient activity of the lysosomal enzyme iduronate-2-sulfatase [1,2]. This deficient activity of iduronate-2-sulfatase results in progressive accumulation of glycosaminoglycans (GAGs) in tissues and organs throughout the body and subsequent multiorgan dysfunction [1,[3], [4], [5], [6]]. Heparan sulfate (HS) and dermatan sulfate are the major GAGs that accumulate in patients with MPS II [7]. MPS II is associated with a broad spectrum of multisystemic symptoms, and there is considerable heterogeneity between patients in the age of presentation, number and type of manifestations, and disease progression [1,3,[8], [9], [10], [11]]. Somatic manifestations of the disease are present in all patients with MPS II and can include coarse facial features, hepatosplenomegaly, joint and skeletal abnormalities, pulmonary dysfunction, and cardiovascular disease [3,8,10]. Approximately two-thirds of patients experience profound cognitive impairment (neuronopathic disease); in these patients, neurodevelopmental delays typically appear between 2 and 4 years of age and progress over time such that patients are not expected to survive past the second decade of life [1,3,[11], [12], [13], [14], [15]]. Cognitive impairment observed in patients with the neuronopathic form of MPS II correlates with elevated HS in the cerebrospinal fluid when compared with healthy controls [16]. Deposition of HS in the brain underlies the progressive neurodegeneration in the neuronopathic forms of MPS II [17]. Patients with non-neuronopathic disease (who do not have progressive neurodegeneration) may present with extra-CNS symptoms later than patients with neuronopathic disease, and may survive beyond the second decade of life [1,3,8,9,18]. The current standard of care for patients with MPS II is intravenous (IV) enzyme replacement therapy (ERT) with recombinant human idursulfase [[19], [20], [21], [22], [23]]. IV idursulfase (Elaprase; Takeda Pharmaceuticals USA, Inc., Lexington, MA, USA) has been shown to alleviate or stabilize a range of somatic manifestations of MPS II [19,20]; however it is not expected to affect CNS disease because it does not cross the blood–brain barrier in therapeutic quantities [[24], [25], [26]]. Treatment strategies that target the CNS disease are under investigation and include intracerebroventricular administration of ERT; conjugation of recombinant enzyme with an endogenous protein, typically a peptide or monoclonal antibody, which enables receptor-mediated transport across the blood–brain barrier; and gene therapy [25,[27], [28], [29], [30], [31], [32]].

To date, limited data are available for determining whether patients with non-neuronopathic MPS II present with different somatic manifestations or undergo different surgical and nonsurgical procedures than those with neuronopathic disease. The Hunter Outcome Survey (HOS; NCT03292887; funded by Takeda Pharmaceutical International AG) is a global, multicenter, observational registry established in 2005 that collects long-term data on the natural history of MPS II and treatment with IV idursulfase [10,21]. The present analysis of HOS data was performed to compare the clinical manifestations, surgical and nonsurgical procedure history, and causes of death in patients with neuronopathic MPS II and in patients with non-neuronopathic MPS II.

## 2 Materials and methods

### 2.1 HOS registry design

HOS is a registry designed for the collection of a broad range of demographic, disease, and treatment-related data on individuals with MPS II during routine patient visits and assessments [10,21]. Male and female patients with a confirmed biochemical or genetic diagnosis of MPS II, including those who are untreated, those receiving treatment with IV idursulfase, and those who received a hematopoietic stem cell transplant, are eligible for enrollment in HOS [10,21]. Patients receiving ERT other than IV idursulfase and patients enrolled in an interventional clinical trial are not eligible. Patient visits and assessments occur as part of routine care with the treating physician; there are no predetermined assessments directed by HOS protocol.

There are guidelines regarding data entry into the HOS database, as specified in the registry protocol. Data can be entered for patients who are either alive at enrollment (prospective patients) or deceased at enrollment (retrospective patients), if local regulations permit. There is no predefined sample size, and any data analyses from HOS are exploratory. Independent Review Board/Ethics Committee approval is obtained for all participating centers before enrollment, and written informed consent is obtained from each patient, their parents, or their legal representative. All patient information is managed in accordance with national data protection standards.

### 2.2 Patient population and data analysis

The prospective patient population for this analysis was identified in a HOS data extraction on 23 July 2018 and included both neuronopathic and non-neuronopathic patients with stable cognitive status defined and confirmed by the principal investigator; patients who had received a bone marrow transplant were excluded. Further data on the previously identified patients were collected from a data extraction conducted on 23 July 2021. No additional patients from this later extraction were included; the analysis was limited to those initially identified in 2018 to verify that the categorizations (e.g., for surgical and nonsurgical procedures) developed based on these patients remained appropriate.

The presence of cognitive impairment was defined from the last available assessment by a healthcare professional and was based on the answer to the following question: Cognitive impairment? Yes/No. Neither the specific assessments that led to the diagnosis of the cognitive impairment nor the degree of cognitive impairment was recorded. Patients with defined cognitive status were identified from those with assessments available at 5, 10, or 9 to 12 years of age and with at least one known follow-up assessment between 1 and 14 years later (Table 1; Suppl Table 1). The different age cutoffs and periods of follow-up were assessed to identify those that provided the optimal balance between a high rate of stability in cognitive status and a high number of patients in a clinically relevant age group. A cutoff age of 10 years with follow-up at 11 to <20 years was selected because changes in cognitive status were infrequent after this, with cognitive impairment status being stable in 151/193 patients, and because it allowed inclusion of a sufficiently large population for analysis of patients in an age group of clinical interest (*n* = 151).

**Table 1:**
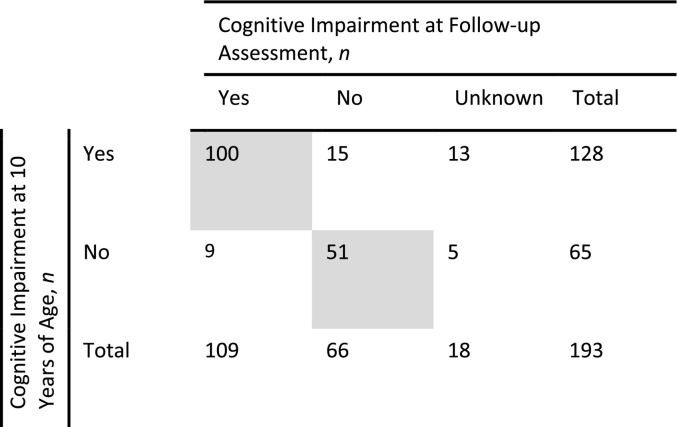
Cognitive impairment of patients with MPS II assessed at 10 years of age and at follow-up visit (at 11 to <20 years of age) in HOS.

Gray shading indicates patients included in the current analysis (*n* = 151).

HOS, Hunter Outcome Survey; MPS II, mucopolysaccharidosis II.

The 151 patients in this selected population were stratified by cognitive impairment status assessed at 10 years of age into neuronopathic (cognitive impairment) and non-neuronopathic (no cognitive impairment) groups. Cases in which the cognitive impairment status changed at a follow-up assessment after the age of 10 years (from “yes” to “no” or from “no” to “yes” in answer to the question “Cognitive impairment?”) or was unknown at follow-up were queried with sites, and any updated information that indicated a change in status was incorporated into the analysis data set. If no further information was available and status remained changed or unknown, these patients were excluded from the analysis. Data from HOS on key parameters, including medical history, demographics, clinical manifestations, history of surgical and nonsurgical procedures, and causes of death were analyzed for patients in the neuronopathic and non-neuronopathic groups. Functional classification, assessed at the last visit, was based on the clinical impression of the attending physician and was recorded as normal (intelligence quotient [IQ] of approximately >70), mild/moderate (IQ of approximately 30–70), or severe/profound (IQ of approximately <30). These categories correspond to, but are not identical to, the severity levels for intellectual disability described in *Diagnostic and Statistical Manual of Mental Disorders*, fourth edition (DSM-IV) [33].

All available data were assessed; no age limit was applied for the collection of data on clinical manifestations and procedures in the two groups.

### 2.3 Clinical manifestations of MPS II

Clinical manifestations of MPS II are recorded using predefined fields within the HOS database. Patients were assumed not to have a sign or symptom of MPS II unless reported otherwise. For this analysis, manifestations of MPS II recorded before and after patients' 10th birthdays were analyzed for the neuronopathic and non-neuronopathic groups and summarized by system organ class. Data on clinical manifestations were grouped as per the preexisting database fields in HOS, with the following exceptions, which were based on the clinical judgment of four of the five authors (H.L., P.H., J.A., B.L.): gait abnormality was reclassified from the neurological system organ class to the musculoskeletal system organ class; two pooled categories were created in the musculoskeletal organ class for joint stiffness in the upper body (including joint stiffness in the hand, elbow, shoulder, wrist, and spine) and joint stiffness in the lower limbs (including joint stiffness in the knee, ankle, and hip).

### 2.4 Surgical and nonsurgical procedures

The type and date of surgical and nonsurgical procedures are recorded for each patient in the HOS database. Patients were assumed not to have undergone any procedure unless reported otherwise. Procedures for which the dates were unknown were assumed to have occurred before each patient's 10th birthday to minimize loss of data and potential bias toward an overestimate of the numbers of procedures in older patients with non-neuronopathic disease. Procedures not covered by the main database fields could be recorded using free text in the “other” category in the HOS database.

As a result of changes to the understanding of intervention patterns in the care of patients with MPS II since the database was established, the procedures were reclassified for this analysis into an updated selection of categories, agreed upon by three of the authors [H.L. P.H., B.L], to capture more accurately and fully all of the different procedures these patients underwent, including those initially recorded using free text in the “other” category. The reclassified categories for surgical and nonsurgical procedures, detailed in full in Suppl Table 2, were abdominal, including gastrostomy, percutaneous endoscopic gastrostomy tube insertion, and hernia repair; cardiac, including valve replacement and pericardial effusion removal; CNS, including intracranial shunt placement/replacement; dental; diagnostic; ear, nose, and throat (ENT), including tracheotomy; foot, including Achilles lengthening; hand and upper limb, including carpal tunnel syndrome (CTS) decompression and trigger finger surgery; health maintenance; hip, including pelvic osteotomy; knee, including knee arthroscopy; port-a-cath placement/replacement and central intravenous line; other; and unknown. Entries were excluded from this analysis if the procedure was considered to be unrelated to MPS II, or if it was not clear that any procedure had taken place. In total, 15 entries were excluded and are detailed in Suppl Table 3. When an entry indicated that two procedures had occurred, this was considered a single procedure in this analysis if both were performed in the same part of the body, for the same reason, and on the same day. Data on the proportion of patients with different procedures recorded before and after their 10th birthdays were analyzed for patients with neuronopathic disease and for patients with non-neuronopathic disease.

### 2.5 Causes of death

The date and cause of death are recorded in the HOS database. In cases when the cause of death is not covered by the main database fields, it may be recorded using free text under the “other” category. The free text was reviewed by the HOS Biostatistician, who is also one of the authors (J.B.), and the HOS Medical Monitor. If the cause of death was considered to fall under a main database category, deaths were reclassified from “other” to the appropriate named category for cause of death. Causes of death were analyzed for patients with neuronopathic disease and for patients with non-neuronopathic disease.

### 2.6 Descriptive statistics and data analysis

Descriptive statistics were determined to summarize the data; median values (10th percentile [P10], 90th percentile [P90]) were calculated unless otherwise stated. The proportion of patients demonstrating MPS II manifestations and undergoing procedures in the neuronopathic and non-neuronopathic groups both before and after patients' 10th birthdays were determined. The proportion of patients demonstrating manifestations by system organ class and the proportion of patients demonstrating manifestations for which there is a >10 percentage point difference between the two groups were determined. In addition to proportion of different procedure types, the median (P10, P90) number of different procedures per patient and the median (P10, P90) total number of procedures per patient, including multiple procedures of the same types were calculated.

## 3 Results

### 3.1 Patient population and characteristics

As of July 2018, there were 1098 prospective patients enrolled in HOS (those alive at HOS entry and followed prospectively in the registry). Overall, 193 of these patients had a cognitive assessment available at 10 years of age and at least one follow-up assessment between 11 years and <20 years of age. Of these patients, 151 (78.2%) had no change in cognitive impairment status between these assessments (Table 1) and were included in this analysis. Excluded were 24 patients (12.4%) for whom cognitive impairment status changed at the follow-up assessment and 18 patients (9.3%) for whom cognitive status was unknown at follow-up.

At 10 years of age and at follow-up, 100/151 patients (66.2%) had cognitive impairment (neuronopathic group) and 51/151 (33.8%) had no cognitive impairment (non-neuronopathic group). The median age at onset of any manifestations was numerically lower in the neuronopathic group than in the non-neuronopathic group (median [P10, P90], 1.5 [0.3, 3.0] years vs 2.0 [0.3, 4.5] years), as was the median age at diagnosis (median [P10, P90], 3.0 [1.4, 5.3] years vs 4.0 [1.0, 7.4] years) (Table 2). The median (P10, P90) age at first cognitive impairment assessment was 4.7 (1.8, 9.7) years in the neuronopathic group and 6.9 (3.6, 10.2) years in the non-neuronopathic group. Two patients in the neuronopathic group were reported to have normal function (IQ of approximately >70) at the last assessment, while assessments both before and after patients' 10th birthdays consistently recorded cognitive impairment. Two patients in the non-neuronopathic group were reported to have moderate functional impairment (IQ of approximately 30–70) at last assessment, while assessments both before and after patients' 10th birthdays consistently recorded no cognitive impairment.

**Table 2:** Patient characteristics and causes of death in the neuronopathic and non-neuronopathic groups.

	Neuronopathic Group (*n* = 100)	Non-neuronopathic Group (*n* = 51)
Male		
*n* (%)	99 (99.0)	50 (98.0)

Age at Onset of Symptoms, years		
*n*	92	47
Median (P10, P90)	1.5 (0.3, 3.0)	2.0 (0.3, 4.5)
Mean (SD)	1.5 (1.04)	2.2 (1.65)

Age at Diagnosis, years		
*n*	97	50
Median (P10, P90)	3.0 (1.4, 5.3)	4.0 (1.0, 7.4)
Mean (SD)	3.2 (1.53)	4.1 (2.41)

Age at Last Visit, years		
*n*	100	51
Median (P10, P90)	15.7 (12.0, 19.9)	17 (12.6, 21.5)
Mean (SD)	15.8 (3.03)	17.0 (3.24)

Age at First Cognitive Impairment Assessment, years		
*n*	100	51
Median (P10, P90)	4.7 (1.8, 9.7)	6.9 (3.6, 10.2)
Mean (SD)	5.1 (2.92)	6.9 (2.61)

Age at ERT Start, years		
*n*	94	47
Median (P10, P90)	6.5 (2.9, 10.1)	6.7 (2.7, 9.2)
Mean (SD)	6.6 (2.60)	6.4 (2.68)

Functional Classification by Clinical Impression at Last Visit		
*n*	74	41
Normal (IQ >70), *n* (%)	2 (2.7)	39 (95.1)
Mild/Moderate (IQ 30–70), *n* (%)	22 (29.7)	2 (4.9)
Severe/Profound (IQ <30), *n* (%)	50 (67.6)	0 (0.0)
Patients receiving ERT, *n* (%)	94 (94.0)	47 (92.2)

Number of Different Types of Procedures per Patient^a^		
*n*	88	49
Median (P10, P90)	3 (1, 6)	3 (1, 7)

Total Number of Procedures per Patient^a^		
*n*	88	49
Median (P10, P90)	4 (1, 10)	5 (2, 11)

Age at First Procedure, years^a^		
*n*	86	45
Median (P10, P90)	2.5 (0.2, 6.8)	2.2 (0.2, 5.1)
Mean (SD)	3.0 (2.52)	2.5 (1.95)
Deceased, *n* (%)	47 (47.0)	1 (2.0)

Cause of Death, n (%)		
Respiratory Failure	15 (31.9)	0 (0.0)
Cardiac Failure	8 (17.0)	0 (0.0)
Cardiorespiratory Failure	6 (12.8)	0 (0.0)
Infection	6 (12.8)	1 (100)
Renal Failure	1 (2.1)	0 (0.0)
Status Epilepticus or Seizure	1 (2.1)	0 (0.0)
Multiple Organ Failure	1 (2.1)	0 (0.0)
Unknown	9 (19.1)	0 (0.0)

a Only procedures performed before patients' 10th birthdays were included. ERT, enzyme replacement therapy; IQ, intelligence quotient; P10, 10th percentile; P90, 90th percentile; SD, standard deviation.

Most patients were receiving ERT: 94.0% of patients in the neuronopathic group and 92.2% of patients in the non-neuronopathic group. In the neuronopathic group and the non-neuronopathic group, the median (P10, P90) age at the start of treatment was 6.5 (2.9, 10.1) years and 6.7 (2.7, 9.2) years, respectively, and the median (P10, P90) duration of treatment was 123.5 (45.0, 159.6) months and 130.6 (93.0, 163.8) months, respectively.

### 3.2 Clinical manifestations

**Fig. 1:**
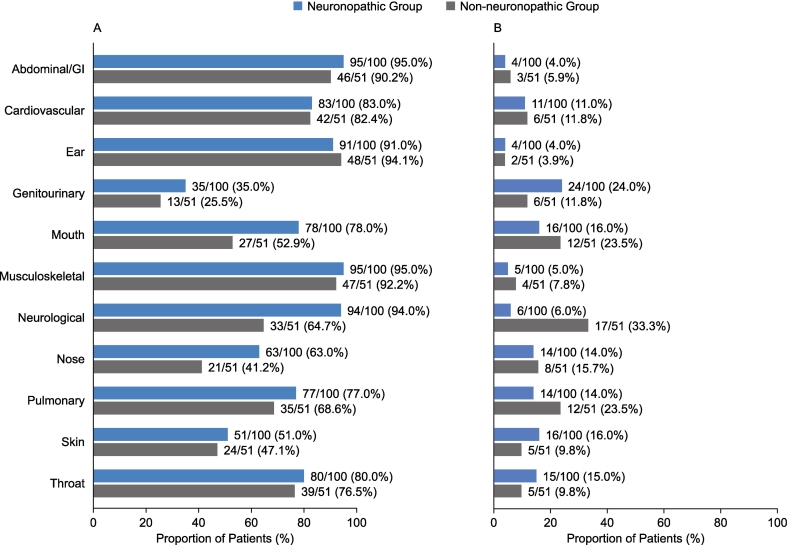
Proportion of patients with somatic and neurological manifestations reported by system organ class in the neuronopathic and non-neuronopathic groups before (A) and after (B) patients' 10th birthdays. GI, gastrointestinal.

**Fig. 2:**
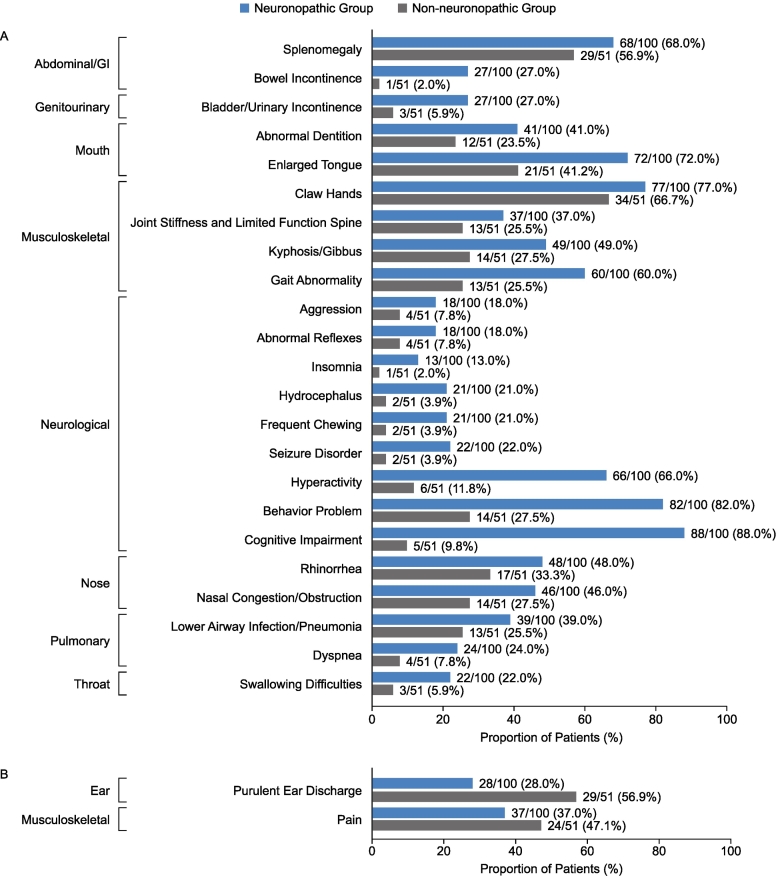
Proportion of patients with somatic and neurological manifestations reported before patients' 10th birthdays with a difference of >10 percentage points between the neuronopathic and non-neuronopathic groups. (A) manifestations more common in the neuronopathic group than in the non-neuronopathic group. (B) manifestations more common in the non-neuronopathic group than in the neuronopathic group. GI, gastrointestinal.

The proportions of patients demonstrating MPS II manifestations reported by system organ classes were broadly comparable for the neuronopathic and non-neuronopathic groups for most categories before (Fig. 1A) and after (Fig. 1B) patients' 10th birthdays. Before patients' 10th birthdays, common manifestations in both the neuronopathic group and the non-neuronopathic group included facial features consistent with MPS II (91.0% and 82.4% of patients, respectively), joint stiffness and limited function (85.0% and 88.2% of patients, respectively), hepatomegaly (87.0% and 80.4% of patients, respectively), hernias (78.0% and 74.5% of patients, respectively), and valve disease (75.0% and 78.4% of patients, respectively). Overall, neurological symptoms, genitourinary symptoms, and symptoms affecting the mouth or nose were reported in a higher proportion of patients in the neuronopathic group than in the non-neuronopathic group before patients' 10th birthdays. Other than cognitive impairment, the specific manifestations occurring in a higher proportion of patients in the neuronopathic group than in the non-neuronopathic group and with the greatest difference between groups were hyperactivity (66.0% vs 11.8% of patients), behavioral problem (82.0% vs 27.5% of patients), gait abnormality (60.0% vs 25.5% of patients), and an enlarged tongue (72.0% vs 41.2% of patients) (Fig. 2A). Only two manifestations with a >10 percentage point difference between groups were reported in a higher proportion of patients in the non-neuronopathic group than in the neuronopathic group before patients' 10th birthdays: purulent ear discharge (56.9% vs 28.0% of patients) and pain (47.1% vs 37.0% of patients) (Fig. 2B).

**Fig. 3:**
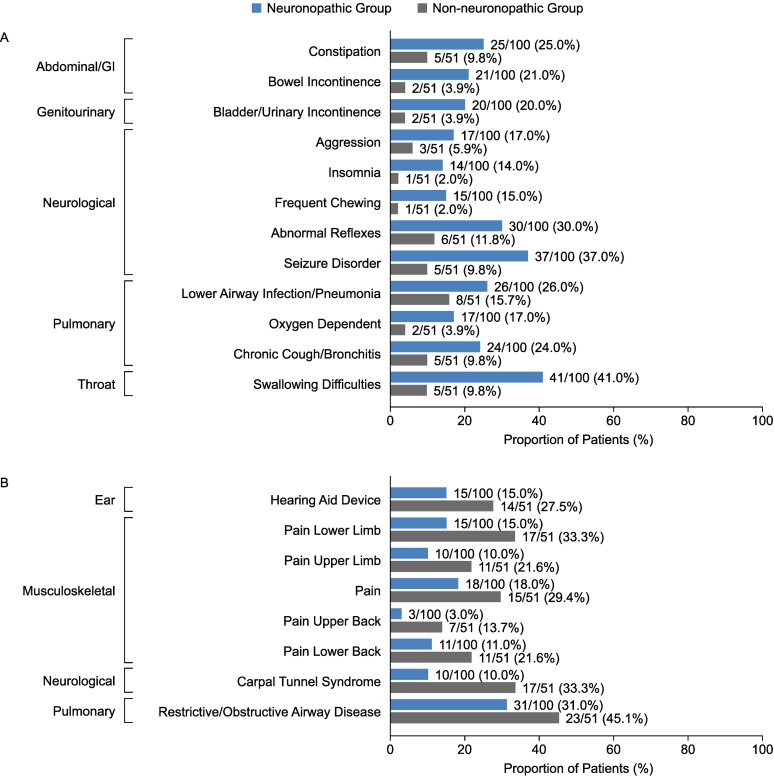
Proportion of patients with somatic and neurological manifestations reported after patients' 10th birthdays with a difference of >10 percentage points between the neuronopathic and non-neuronopathic groups. (A) somatic manifestations more common in the neuronopathic group than in the non-neuronopathic group (B) somatic manifestations more common in the non-neuronopathic group than in the neuronopathic group. GI, gastrointestinal.

After patients' 10th birthdays, the manifestations occurring in a higher proportion of patients in the neuronopathic group than in the non-neuronopathic group with the greatest difference in proportion between groups included swallowing difficulties (41.0% vs 9.8% of patients), seizure disorder (37.0% vs 9.8% of patients), and abnormal reflexes (30.0% vs 11.8% of patients) (Fig. 3A). Manifestations occurring in a higher proportion of patients in the non-neuronopathic group than in the neuronopathic group included CTS (33.3% vs 10.0% of patients), pain lower limb (33.3% vs 15.0% of patients), respiratory/obstructive airway disease (45.1% vs 31.0% of patients), hearing aid device (27.5% vs 15.0% of patients), and pain upper limb (21.6% vs 10.0% of patients) (Fig. 3B).

### 3.3 Surgical and nonsurgical procedures

Most patients in both the neuronopathic and non-neuronopathic groups had undergone surgical or diagnostic procedures before their 10th birthday: 88/100 patients (88.0%) in the neuronopathic group and 49/51 patients (96.1%) in the non-neuronopathic group. In total, 446 procedures were recorded among the 88 patients in the neuronopathic group, and 286 procedures were reported for the 49 patients in the non-neuronopathic group. The median (P10, P90) age at first procedure was 2.5 (0.2, 6.8) years in the neuronopathic group and 2.2 (0.2, 5.1) years in the non-neuronopathic group (Table 2).

**Fig. 4:**
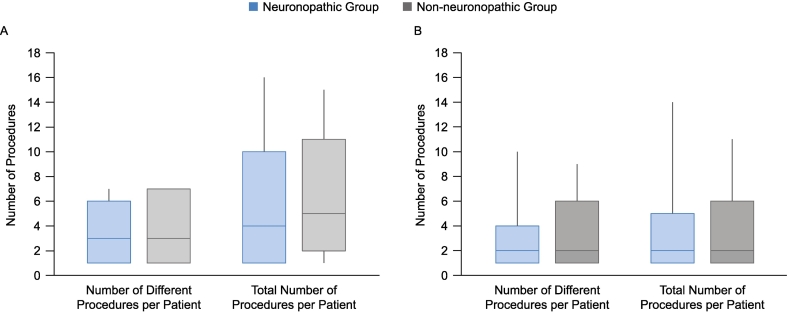
Numbers of different types of procedures per patient and numbers of all procedures per patient before (A) and after (B) patients' 10th birthdays. Median (P10, P90) values are shown, whiskers represent maximum and minimum values.

The median (P10, P90) number of different types of procedures that occurred before the patients' 10th birthdays was the same in both groups: neuronopathic group, 3 (1, 6) per patient; non-neuronopathic group, 3 (1, 7) per patient (Table 2; Fig. 4). The highest number of different procedures that occurred before the age of 10 years was seven per patient, and this was reported for 4.0% and 9.8% of patients in the neuronopathic group and non-neuronopathic group, respectively. The median (P10, P90) number of all procedures that occurred before the patients' 10th birthdays, including multiple procedures of the same type, was comparable in the two groups: neuronopathic group, 4 (1, 10) per patient; non-neuronopathic group, 5 (2, 11) per patient.

**Fig. 5:**
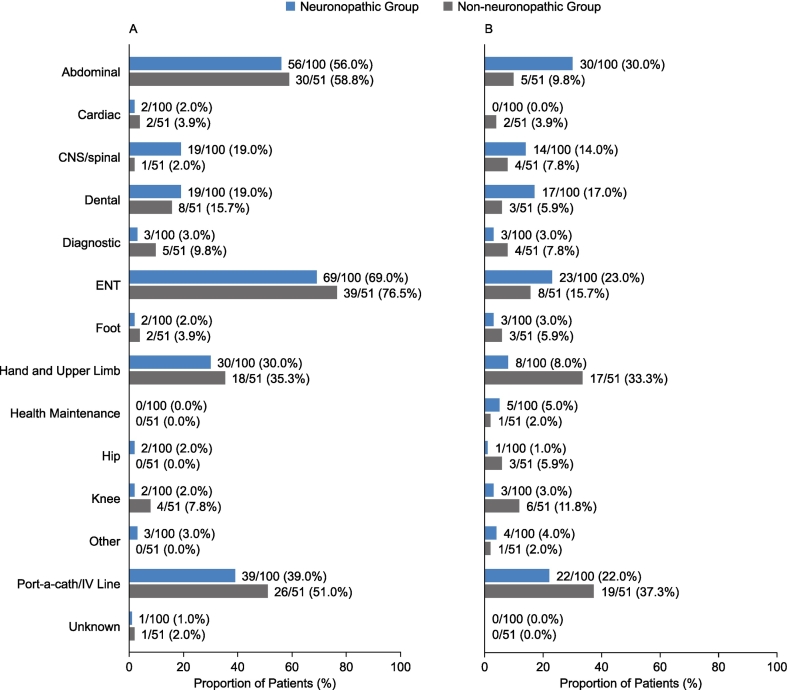
Proportion of patients undergoing surgical and nonsurgical procedures by category in the neuronopathic and non-neuronopathic groups before (A) and after (B) their 10th birthdays. Details of the procedure categories are given in Suppl. Table 2. CNS, central nervous system; ENT, ear, nose, and throat; IV, intravenous.

The proportion of patients who underwent different procedures before their 10th birthday was broadly comparable in the neuronopathic and non-neuronopathic groups for most categories (Fig. 5). The most commonly occurring categories of procedures in both the neuronopathic group and non-neuronopathic group that occurred before patients' 10th birthdays were ENT procedures (69.0% and 76.5% of patients, respectively), abdominal procedures (56.0% and 58.8% of patients, respectively), and port-a-cath/central intravenous line procedures (39.0% and 51.0% of patients, respectively) (Fig. 5A). Other procedures recorded for a higher proportion of patients in the non-neuronopathic group than in the neuronopathic group before patients' 10th birthdays included diagnostic procedures (9.8% vs 3.0%,), hand and upper limb procedures (35.3% vs 30.0%), and knee procedures (7.8% vs 2.0%). The proportion of patients who underwent CNS procedures before their 10th birthdays was higher in the neuronopathic group than in the non-neuronopathic group (19.0% vs 2.0%). In addition, the proportions of patients who underwent dental procedures, hip procedures, and “other” procedures before their 10th birthdays were slightly higher in the neuronopathic group than in the non-neuronopathic group (19.0% vs 15.7%, 2.0% vs 0.0%, and 3.0% vs 0.0%, respectively).

After patients' 10th birthdays, there were 192 surgeries in total in 67 patients in the neuronopathic group and 109 procedures in 37 patients in the non-neuronopathic group. The median (P10, P90) number of different types of procedures per patient (2 [1,4] and 2 [1,6] in the neuronopathic group and non-neuronopathic group, respectively) and of all procedures, including multiple procedures of the same type, per patient (2 [1,5] and 2 [1,6] in the neuronopathic group and non-neuronopathic group, respectively) were similar between the two groups (Fig. 4).

In both groups, the proportion of patients who underwent different procedures after their 10th birthdays was generally lower than that reported before their 10th birthdays (Fig. 5B). In contrast to before patients' 10th birthdays, the proportions of patients who underwent abdominal procedures and ENT procedures after their 10th birthdays were higher in the neuronopathic group than in the non-neuronopathic group (30.0% vs 9.8% and 23.0% vs 15.7%, respectively) (Fig. 5B). After patients' 10th birthdays, the proportions of patients who underwent CNS procedures and dental procedures remained higher in the neuronopathic group than in the non-neuronopathic group (14.0% vs 7.8% and 17.0% vs 5.9%, respectively), and the proportions of patients who underwent hand and upper limb procedures, port-a-cath/central intravenous line procedures, and diagnostic procedures remained higher in the non-neuronopathic group than in the neuronopathic group (33.3% vs 8.0%, 37.3% vs 22.0%, and 7.8% vs 3.0%, respectively).

### 3.4 Causes of death

Mortality was higher in the neuronopathic group than in the non-neuronopathic group (Table 2). In the neuronopathic group, 47/100 patients (47.0%) died, with the most common recorded causes of death being respiratory failure (31.9%), “unknown” cause (19.1%), cardiac failure (17.0%), and cardiorespiratory failure (12.8%); the median (P10, P90) age of death was 15.4 (12.3, 19.8) years. In the non-neuronopathic group, there was one death, which was due to pneumonia (age at death, 19.8 years).

## 4 Discussion

This analysis of data from HOS demonstrates the considerable clinical burden of both neuronopathic and non-neuronopathic forms of MPS II. The proportion of patients demonstrating clinical manifestations and undergoing surgical and nonsurgical procedures was similar between patients with non-neuronopathic MPS II and patients with neuronopathic MPS II.

In HOS, the absence or presence of cognitive impairment is recorded as the answer given to the supplied question “Cognitive impairment? Yes/No” and may be based on the results from standardized cognitive tests or the impression of the treating physician. This results in a lack of standardization across sites and physicians. Given that the answer to this question was used to categorize patients into the two comparator groups in the current analysis, namely, those with neuronopathic MPS II and those with non-neuronopathic MPS II, the lack of standardization may have led to inconsistencies in patient assignment to the two categories. However, this approach for assessing cognitive impairment most likely reflects real-life practice and may overcome some of the recognized limitations of cognitive tests. The validity and reliability of commonly used cognitive tests, such as the Differential Abilities Scale, second edition, may be affected by somatic, motor, sensory, and behavioral problems in patients with MPS II; training and experience of the examiner; language and cultural issues; and the testing environment [34,35]. The ability of these tests to assess changes in cognitive ability over time may also vary with age and level of cognitive impairment of the patient and remains to be fully established in different populations [36]. In the present analysis, identification of a population of patients with stable cognitive impairment status was required to establish two groups of patients: those with the neuronopathic form and those with the non-neuronopathic form of MPS II. Different ages at the time of cognitive assessments and different periods of follow-up were explored, and the associated stability of cognitive impairment status and size of the resulting populations were evaluated. Several studies of the natural history of neuronopathic MPS II have reported an onset of cognitive impairment at 2–4 years of age in many patients, while another study demonstrated onset of neurodegeneration between 4 years and 10 years of age [15,[37], [38], [39], [40]]. In the current study, the population of patients who had a cognitive assessment at 10 years of age and then a follow-up assessment at 11 to <20 years of age had a relatively high rate of stability of cognitive impairment status (78%) and provided 151 patients with MPS II and stable cognitive impairment status for inclusion in the analysis. This criterion resulted in the inclusion of the small number of patients in this analysis who had stable assessments of cognitive impairment that did not appear to be consistent with the functional classification at last visit. These included the two patients who were classed as having non-neuronopathic disease but were recorded as having mild/moderate functional impairment (low IQ) at last assessment. It is possible that these patients experienced developmental delays or behavioral features as a result of chronic disease and not as a manifestation of CNS involvement. Also included were the two patients who were classed as having neuronopathic disease but for whom normal cognitive function was recorded at last assessment. These patients were included because the results of their cognitive impairment assessment was stable. Differences in the timing of and methodology for the assessment of cognitive impairment and functional classification may explain the discrepancies in our findings. Interestingly, nine patients were excluded from this analysis who demonstrated absence of cognitive impairment at 10 years of age but subsequently received a diagnosis of cognitive impairment at follow-up assessment. Although the presence of CNS involvement is conventionally described as a severe phenotype with early progression [27], these nine patients may represent a subpopulation with a more gradual onset neuronopathic phenotype.

For this analysis, somatic manifestations of MPS II were demonstrated in a broadly similar proportion of patients with neuronopathic or non-neuronopathic MPS II. The most common manifestations before patients' 10th birthdays, occurring in ≥80% of patients in one or both groups, included abdominal/gastrointestinal, cardiovascular, musculoskeletal, neurological, ear, and throat categories, and for these the difference in proportion of patients between the two groups was <5% for all except the neurological category. Previous reports have also described similar somatic involvement in the non-neuronopathic form of MPS II and the neuronopathic form, albeit with a reduced rate of progression [1,3,8,37,41]. This contrasts with the somatic presentation of mucopolysaccharidosis VI, another non-neuronopathic mucopolysaccharidosis, which differs markedly between slowly and rapidly progressive forms [39]. These findings suggest that the presence or absence of cognitive impairment is not predictive of the somatic disease burden.

As expected, neurological manifestations were notably more common in the neuronopathic group than in the non-neuronopathic group before patients' 10th birthdays. Other features and consequences of MPS II, including fatigue, sleep disturbances, and psychological impacts of chronic illness may also have influenced neurological manifestations recorded for the neuronopathic group [35,42,43].

Manifestations affecting the nose and mouth were also considerably more common in the neuronopathic group than in the non-neuronopathic group before patients' 10th birthdays, with a between-group difference in proportion of patients of approximately 22% (nose) and 25% (mouth). In an early description of the neuronopathic form of MPS II, mouth and nose manifestations such as an enlarged tongue, abnormal dentition, and rhinorrhea were reported in most if not all patients [12]. The only two manifestations more common in the non-neuronopathic group than in the neuronopathic group were purulent ear discharge and pain. It is possible that symptoms such as pain may have been underreported in the neuronopathic group owing to patients being nonverbal. These symptoms may manifest in other symptom categories, for example as increased behavioral difficulties, and/or have an impact in delaying and subsequently reducing the success of procedures owing to the inability of patients to articulate symptoms or cooperate.

The proportion of patients demonstrating different manifestations in both the neuronopathic and non-neuronopathic groups was lower after the patients' 10th birthdays than before them. The unknown age distribution of patients at their last follow-up may have influenced this difference however, and contributed to the lower recorded proportion after patients' 10th birthdays. Certain manifestations, such as pain and CTS, may be underreported in older patients with neuronopathic disease owing to communication difficulties but may also affect externally diagnosed manifestations such as behavioral problems.

The finding that 88% and 96% of patients in the neuronopathic group and non-neuronopathic group, respectively, underwent a surgical or diagnostic procedure before their 10th birthday is in line with previous reports of 79–91% of patients with MPS II having undergone at least one surgical procedure [6,[44], [45], [46]]. In a study of patients treated in the French healthcare system (*n* = 52), the proportion of patients who had undergone at least one surgical procedure was reported to be similar in patients with neuronopathic MPS II and those with non-neuronopathic MPS II [46]. In the current analysis (*n* = 151), in addition to the overall proportion of patients having undergone surgical and nonsurgical procedures, the number of different types of procedures per patient and the total number of procedures per patient were comparable in the neuronopathic and non-neuronopathic groups before and after patients' 10th birthdays.

Since 2005, when HOS was first established, our knowledge on and experience with all aspects of MPS II, including the associated surgical and nonsurgical procedures, have grown considerably. Thus, it seemed appropriate to reclassify the procedures documented in HOS to reflect all this gained knowledge and experience. With the updated categories, “other” procedures were recorded for ≤4% of patients in this study compared with 29% of patients in a previous HOS analysis [44]. Any classification system, however, will inevitably have its limitations, particularly in the case of a disease for which there is still much to learn. For example, multiple entries on procedures were excluded based on the lack of an established association with MPS II pathology, but such associations may yet be determined.

The differences in proportions of patients undergoing most types of surgical and nonsurgical procedures between the neuronopathic and non-neuronopathic groups were small. The most common procedures in both groups before patients' 10th birthdays were in the ENT, abdominal, and port-a-cath/intravenous line categories, in agreement with previous reports [6,[44], [45], [46]]. For each of these three categories, the proportion of patients with a history of the procedure was higher in the non-neuronopathic group than in the neuronopathic group. Similarly, in a previous report, the proportion of patients with a history of adenoidectomy and tonsillectomy was higher in those with the non-neuronopathic form than in those with the neuronopathic form [46]. The higher proportion of diagnostic procedures before patients' 10th birthdays in the non-neuronopathic group than in the neuronopathic group may reflect the challenge of diagnosing the non-neuronopathic form, owing to the lack of cognitive impairment and a more gradual onset of less rapidly progressing, non-specific manifestations [47,48]. However, different challenges impact diagnoses of the neuronopathic form of MPS II, owing to associated communication and language issues [42]. Hand and upper limb procedures, which included CTS decompression, were also more frequent in the non-neuronopathic group than in the neuronopathic group, as may be expected from the reported higher proportion of patients with the non-neuronopathic form demonstrating CTS [1,3,41]. The greater proportion of patients undergoing CNS/spinal procedures in the neuronopathic group compared with the non-neuronopathic group was also as expected given the characteristic neurological decline in the neuronopathic form [1,25,41].

The number of different procedures per patient and the total number of procedures per patient remained comparable in the neuronopathic and non-neuronopathic groups after patients' 10th birthdays, with the proportion of patients undergoing most types of procedures being lower after patients' 10th birthdays than before them in both groups. Two common procedure categories, abdominal procedures and ENT procedures, became more prevalent in the neuronopathic group than in the non-neuronopathic group after patients' 10th birthdays, in contrast to the findings before patients' 10th birthdays. This may be a result of the more rapid deterioration of patients with the neuronopathic form in the second decade of life [1,3]. As discussed previously, however, the data after patients' 10th birthdays must be interpreted with caution.

The number of times patients undergo anesthesia is also an important consideration, and it is likely that there is a greater need for anesthesia in patients with more severe manifestations. As a result of the pulmonary, upper respiratory tract, musculoskeletal, abdominal, cardiac, and neurological manifestations of MPS II, risks associated with anesthetics are increased [3,44,49,50]. In a study of anesthesia in patients with mucopolysaccharidoses, 3 (43%) of 7 patients with MPS II had difficult or failed intubations [49]. Complications from the anesthesia can lead to additional emergency procedures being carried out and even death [3,44,50,51]. While use of anesthesia is not specifically collected in HOS, analysis of the total number of procedures per patient allowed some accounting for the number of times patients underwent anesthesia. Results for the total number of procedures per patient were similar in the neuronopathic and non-neuronopathic groups. However, the equivalence between number of procedures and times under anesthesia is an approximation, because consequences of MPS II such as pain, cognitive impairment, and behavioral problems may influence the necessity for the use of anesthetics during certain surgical/interventional procedures, such as some dental procedures.

Over 90% of patients in both the neuronopathic and non-neuronopathic groups were receiving the standard of care, namely, ERT. Age at treatment initiation and duration of treatment were also comparable in the two groups. Thus, treatment with ERT is unlikely to be a confounding factor in this analysis.

The demonstration of a similar burden of significant somatic disease and surgical and nonsurgical procedures in patients with either form of MPS II lends further support to the consideration of this disease as having a spectrum of severity rather than two types/categories of severity. While two distinct forms of MPS II emerged in early studies, based on disease severity, the presence of progressive cognitive impairment, and patient lifespan, the current increase in evidence and knowledge base suggests that these forms are two extremes of a spectrum, that multiple intermediate phenotypes may exist, and that patients with a diagnosis of the non-neuronopathic form may have a significant somatic disease burden leading to significant morbidity and disability [3,15,37,39,41]. In addition, the present results further highlight the need for early diagnosis and intervention and optimized management of patients with non-neuronopathic MPS II.

There are several limitations to the current analysis that should be considered. This is a nonprospective analysis of data from an observational registry, with no standardization of methods, techniques, assays, and assessments across sites. There was also a lack of standardization for the recording of cognitive impairment status in HOS, and some patients may have been misclassified in the two groups. While efforts were made to optimize the updated classification approach for surgical and nonsurgical procedures, misclassifications may again have occurred. In addition, robust comparisons between groups for some categories were precluded by small sample size and low event rates. It is possible that pediatric patients were lost to follow-up owing to transition to adult medicine. In addition, differences in the perceived balance of benefits and risks of some procedures for patients with neuronopathic disease and those with non-neuronopathic disease may have affected the results, with potentially greater reluctance among physicians and patients' families to perform some procedures in patients with neuronopathic disease because of potential problems with anesthesia and the more limited life expectancy for these patients. It may also be more difficult to assess the need for some procedures such as carpal tunnel or cervical decompression surgery in patients with neuronopathic disease as a result of challenges in communication and cooperation. Furthermore, as highlighted above, caution is required when interpreting the data recorded after patients' 10th birthdays owing to unknown age distribution for the final follow-up and differences in mortality between the two groups.

## 5 Conclusions

This HOS analysis demonstrates the substantial disease burden associated with both neuronopathic and non-neuronopathic MPS II. Patients with non-neuronopathic disease have a complex array of manifestations and undergo surgical and nonsurgical procedures within the first two decades of life as often as those with neuronopathic disease. This highlights the importance of close monitoring and of timely and comprehensive clinical intervention in this group of patients. The terms “attenuated”, “mild”, and “slowly progressing” may be misleading for describing patients with non-neuronopathic MPS II given the significant manifestations they experience and procedures they undergo in the first two decades of life.

## Ethics approval and consent to participate

Independent Review Board/Ethics Committee approval was obtained for all participating centers. HOS is conducted in accordance with Good Pharmacoepidemiological Practices (GPP), Good Research for Comparative Effectiveness principles, and the relevant principles of the International Conference on Harmonisation (ICH) Good Clinical Practice (GCP) guidelines (ICH E6). Each patient or their parents or a legal representative provided signed-and-dated written informed consent for participation in HOS. All patient information is managed in accordance with national data protection standards.

## Consent for publication

Each patient or their parents or a legal representative provided informed consent for publication of data.

## Funding

This study was funded by Takeda Pharmaceuticals International AG. Collection and analysis of data in HOS is supported by Takeda Pharmaceuticals International AG and data analyses were performed by Takeda Pharmaceuticals International AG under the direction of the authors. Medical writing assistance, provided under the direction of the authors, was funded by Takeda Development Center Americas, Inc.

## Declaration of Competing Interest

Heather Lau has received consulting fees from Amicus Therapeutics, Pfizer, Sanofi Genzyme, Takeda, and Taysha and research grants from Amicus Therapeutics, BioMarin, Pfizer, Protalix Biotherapeutics, Sangamo Therapeutics, Sanofi Genzyme, Takeda, and Ultragenyx, and is a full-time employee and stockholder of Ultragenyx Pharmaceutical. Paul Harmatz has received honoraria, consulting fees, and/or research grants from Adrenas Aeglea, Allievex, Amicus Therapeutics, Ascendis Pharma, Aspa therapeutics, Astellas Gene Therapies, AVROBIO, Azafaros, BioMarin, Calcilytix, Capsida Biotherapeutics, Chiesi, Denali Therapeutics, EdiGene, Grace Science, Homology Medicines, Inventiva Pharma, JCR Pharmaceuticals, Novel Pharma, Orchard Therapeutics, Orphazyme, Paradigm, QED Therapeutics, RallyBio, REGENXBIO, Renoviron, Sangamo Therapeutics, SalioGen Therapeutics, Sanofi Genzyme, Sobi and Takeda. Bianca Link has received fees and travel grants from Alexion, Sanofi Genzyme, Shire, and Takeda. Jaco Botha is a full-time employee of Takeda Pharmaceuticals International AG and stockholder of Takeda Pharmaceutical Company Limited. Jennifer Audi was a full-time employee of Takeda Pharmaceuticals International AG (now with Ultragenyx Europe GmbH) and was a stockholder of Takeda Pharmaceutical Company Limited.

## Data Availability

The data sets, including redacted study protocol, redacted statistical analysis plan, and data for individual participants that support the results reported in this article, will be made available within 3 months from initial request to researchers who provide a methodologically sound proposal. The data will be provided after its deidentification in compliance with applicable privacy laws, data protection, and requirements for consent and anonymization.

## Appendix A Supplementary data

Supplementary material Image 1
