# Supplementary material for: Clinical characteristics and somatic burden of patients with mucopolysaccharidosis II with or without neurological involvement: An analysis from the Hunter Outcome Survey
Source: Mol Genet Metab Rep. 2023 Sep 8;37:101005. doi: 10.1016/j.ymgmr.2023.101005 (PMC10694755; doi:10.1016/j.ymgmr.2023.101005)
Supplement: Supplementary file 1 — Supplementary material [file mmc1.docx]

**Supplementary file**

**Clinical characteristics and somatic burden of patients with mucopolysaccharidosis II with or without neurological involvement: an analysis from the Hunter Outcome Survey** Heather Lau, Paul Harmatz, Jaco Botha, Jennifer Audi, Bianca Link

**Supplementary Table 1**

Cognitive impairment in patients who have an assessment reported at 5 years of age (A) or 9 to <12 years of age (B) and at least one subsequent assessment between 10 years of age (A) or 12 years of age (B) and <20 years of age.

A

|  | | Cognitive Impairment at Follow-up Assessment  (10 to <20 years of age at last visit), *n* | | | |
| --- | --- | --- | --- | --- | --- |
|  |  | Yes | No | Unknown | Total |
| Cognitive Impairment at 5 Years of Age, *n* | Yes | 77 | 16 | 7 | 100 |
|  | No | 10 | 41 | 1 | 52 |
|  | Total | 87 | 57 | 8 | 152 |

B

|  | | Cognitive Impairment at Follow-up Assessment  (12 to <20 years at last visit), *n* | | | |
| --- | --- | --- | --- | --- | --- |
|  |  | Yes | No | Unknown | Total |
| Cognitive Impairment at 9 to <12 Years of Age, *n* | Yes | 99 | 13 | 18 | 130 |
|  | No | 5 | 57 | 4 | 66 |
|  | Total | 104 | 70 | 22 | 196 |

Gray shading indicates the number of patients whose cognitive status remained unchanged between earlier and subsequent assessments.

**Supplementary Table 2**

Summary of procedure classifications.

| Reclassified Category | Data entry type | Included Procedures |
| --- | --- | --- |
| Abdominal Procedures, Including Gastrostomy PEG Tube Insertion and Hernia Repair | CRF Categories | Appendectomy, gastrostomy PEG tube insertion, hernia repair |
|  | “Other” Procedures | Cholecystectomy, epicystostomy, fundoplication, hydrocele repair/correction, operation on hydrocele sac, repair of undescended left testicle, retentio testis |
| Cardiac Procedures, Including Valve Replacement and Pericardial Effusion Removal | CRF Categories | Valve replacement/repair |
|  | “Other” Procedures | Aortic root replacement, removal of pericardial effusion |
| CNS and Spinal Procedures, Including Intracranial Shunt Placement/Replacement and Spinal Decompression and Fusion | CRF Categories | Cervical decompression, cervical fusion, cervicolumbar fusion, IDDD placement/replacement, intracranial shunt placement/replacement |
|  | “Other” Procedures | Back surgery to remove metal implants, burr evacuation of subdural hematoma, corporectomy L2, EVD for ICP monitor and drainage of IVH, fusion L2–L3 pelvis, spinal decompression, subdural hematoma surgery, vertebral arthrodesis T11–L4 |
| Dental Procedures | CRF Categories | Dental procedures |
|  | “Other” Procedures | None |
| Diagnostic Procedures | CRF Categories | None |
|  | “Other” Procedures | Auditory brain stem response for hearing, bone marrow aspiration, bronchoscopy, colonoscopy, EUA ears, EUA/arthrogram of both hips, intraesophageal pH monitoring study, laryngoscopy, lumbar spinal tap/LP |
| ENT Procedures, Including Tracheotomy | CRF Categories | Adenoidectomy, ear tube insertion, tonsillectomy, tracheotomy |
|  | “Other” Procedures | Adenotonsillectomy, bilateral submandibular gland resection, cholesteatoma excision, ear paracentesis, endotracheal stent implantation, induction/intubation, insertion of grommets, myringoplasty, nasal stents, otological microsurgery, pharyngeal lesion exeresis, removal of foreign body at carina, resection of prolapsing arytenoid, revision adenoidectomy, tracheostomy tube first insertion, tympanoplasty, uvulopalatopharyngeal surgery |
| Foot Surgeries, Including Achilles Lengthening | CRF Categories | Achilles lengthening |
|  | “Other” Procedures | Emmert plasty, foot deformity surgery, foot osteotomy, foot surgery |
| Hand and Upper Limb Surgeries, Including Carpel Tunnel Syndrome Decompression and trigger finger surgery | CRF Categories | Carpal tunnel decompression, trigger finger surgery |
|  | “Other” Procedures | Intramedullary fixation right humerus, panaritium left hand, right wrist ganglion aspiration, ulnar nerve decompression |
| Health Maintenance Procedures | CRF Categories | None |
|  | “Other” Procedures | Catheter extraction, change of tracheostomy tube, DLTB, gastrostomy tube placement/change/removal, nasogastric tube insertion, percutaneous endoscopic gastrostomy, urinary catheter insertion |
| Hip Procedures, including pelvic osteotomy | CRF Categories | Hip osteotomy, hip replacement, pelvic osteotomy |
|  | “Other” Procedures | Application of internal fixation to bone neck, articular hip puncture, right acetabuloplasty surgery |
| Knee Procedures, Including Knee Arthroscopy | CRF Categories | Femoral osteotomy, knee arthroscopy |
|  | “Other” Procedures | Bilateral correction to valgus knees, bilateral proximal tibial medial physeal figure of, epiphyseal stapling of knees, femoral epiphysiodesis (bilateral), hemiepiphysiodesis bilateral of the knees, insertion and removal of eight-plates, knee other, osteosynthesis of femoral fracture, staple removal, tibial eight-plates, tibial osteotomy |
| Port-a-cath Placement/ Replacement and Central IV Line | CRF Categories | Port-a-cath placement/replacement |
|  | “Other” Procedures | Central catheter insertion, central venous reservoir, manipulation of central line, partial removal of IVAD, PICC line, reservoir implant, TIVAD |
| Other | “Other” Procedures | Removal of cyst on leg, extraction of splints from legs/metal orthoses extraction, fasciotomy, osteosynthesis with femoral plate, plastic surgery, ventriculectomy (two entries of no free text entered; no further information available) |
| Unknown | CRF Categories | Unknown |

CRF, case report form, DLTB, direct laryngo-tracheo-bronchoscopy; ENT, ear, nose, and throat; EUA, examination under anesthetic; EVD, external ventricular drain; ICP, intracranial pressure; IDDD, intrathecal drug delivery device; IV, intravenous; IVAD, implantable venous access device; IVH, intraventricular hemorrhage; L, lumbar vertebra; LP, lumbar puncture; PEG, percutaneous endoscopic gastrostomy; PICC, peripherally inserted central catheter; T, thoracic vertebra; TIVAD, totally implantable venous access device.

**Supplementary Table 3**

Excluded procedure entries.

| Excluded Entries |
| --- |
| Ankyloglossia reduction at 24 months of age |
| Bilateral orchiectomy |
| Circumcision, circumcision revision |
| Exeresis, Meckel's diverticulum onepigastrichernia |
| Frenuloplasty |
| Infection central venous reservoir |
| Kyphosis |
| Neuromuscular left-convex lumbar scoliosis |
| Palmaris longus to extensor pollicis brevis tendon |
| Phimosis correction |
| Placement of insulin pump because of diabetes mellitus type 1 |
| Plantar fascia |
| Polydactylia of left foot |
| Spleen fracture |
| Tenoligamptocapsulotomy |
